# Following the organism to map synthetic genomics

**DOI:** 10.1016/j.biotno.2022.07.001

**Published:** 2022-07-14

**Authors:** Maya Hey, Erika A. Szymanski

**Affiliations:** Colorado State University, Department of English, USA

**Keywords:** Synthetic genomics, Parts-to-whole assembly, Organisms, Model organisms, Responsible research and innovation

## Abstract

Synthetic genomics, or engineering biology at the level of whole genomes and whole organisms, is an emerging outgrowth of parts-based synthetic biology. This nascent subfield is also diverse and difficult to characterize. As social scientists investigating responsible research and innovation in synthetic genomics, we suggest that focusing on the organism is a fruitful approach to making sense of the diversity it encompasses. Here, we offer a heuristic in the form of a tagging system to organize projects by the roles the engineered organism is asked to perform. We suggest several reasons why this system is useful for understanding the current shape and future directions of the field, especially in light of the need to ask: how does engineering biology contribute to building a future of sustainable relationships with other creatures?

## 1 Introduction

### 1.1 The emerging subfield of synthetic genomics

Synthetic biology has its roots in an analogy between DNA and computer code, assembling genes and pathways to be loaded into cellular operating systems.^1^ This parts-based approach remains synthetic biology's core. But, as synthetic biology continues to develop, the idea of designing and building with genetic material is being aimed at larger targets, including engineering in the context of whole genomes and whole organisms.^2^

Whole-genome engineering seems to be an emerging field, but describing it as one is difficult because approaches to synthetic genomes vary widely in terms of the technologies being employed.^3^, ^4^, ^5^, ^6^ While some projects are organized around wholes—whole pathways, whole chromosomes, whole genomes—many of these are targeted through parts, blurring boundaries with conventional synthetic biology. Rather than describing the field in terms of the techniques applied to engineering genomes,^7^ we suggest that one strategy for making sense of this heterogeneity is to consider the organism itself. We ask: what can the role of the organism tell us about the shift towards genome-based engineering?

### 1.2 Centering the organism in genome-based engineering

To map the variety of genome-centric projects, we focus on the role of the organism rather than the role of particular technologies for several reasons. First, as we have already indicated, engineering techniques do not make visible the distinctions between parts and wholes, which we think are most important to understanding the complexity of this emerging phase of engineering biology. The approaches are sometimes similar, but the motives, agendas, and contexts differ.

Organismal roles, in contrast, point to the rationale for choosing particular organisms for particular purposes and the argument for why the work needs to be done. These justifications help us analyze how engineering at the level of genome brings with it different concerns: among scientific communities and policies, among technologies and their promises, and among humans and the various other organisms upon whose lives ours depend. Finally, an organism-centric view makes patterns visible across the working relationships we have with organisms, while also foregrounding the idiosyncrasies of a particular organism. This enables us to make generalizations without dismissing details.

As genome-level engineering continues to grow and develop, we expect that an organism-centric view will illuminate how synthetic genomics engineers relationships as well as things, in ways that highlight common themes while remaining context-sensitive.

## 2 A heuristic for understanding the role of the organism

We have developed the following heuristic on the basis of document analyses, sense-checked through informal interviews and our team's prior ethnographic experiences in the field. As a heuristic, it leaves space for us to expand it as the field and our knowledge about it grow.

Our heuristic relies on a tagging system rather than a series of bins for two reasons. First, in many cases, an organism's role in a project can be categorized in more than one way. While these roles could be ordered and prioritized, we suspect that projects will bear multiple tags and studying these patterns will be useful. Second, a tagging system is easy to expand as the field grows and changes—maybe as additional ways of working with organisms are innovated—without giving the impression (as a binning system might) of being comprehensive or definitive. And as a helpful bonus, tags do not tend to suggest that any of these ways of working is better or more valuable than any other.

### 2.1 Organismal roles organized by tags

Organism as bioreactor. The organism's role is to produce a compound. It satisfies this role through synthetic genetic pathways that maximize substrate uptake and efficient conversion of that substrate to the target output.^8^ Bioreactor organisms are often treated as chassis, or frameworks for supporting engineered structures.^9^

Organism as expert. The organism excels at some particular function, or has some particular feature or trait for which it can be uniquely exploited. For example, an organism may be an expert in tolerating drought by metabolizing crassulacean acid.^10^ *Saccharomyces cerevisiae* are notable experts in homologous recombination,^11^ and can be called in to perform this function even when they are not themselves the organism of interest. The expert organism may either be employed to perform its signature feat or it may be used to “teach” that feat to other organisms when scientists modularize and transfer the relevant genetic pathways into other organisms.^12^

Organism as dataset. The organism's genome is a (presumably underexplored) resource to be mined for valuable new codes.^13^ These codes can be in the form of gene clusters or pathways that can be manipulated and/or transferred to other (bioreactor) organisms to produce new compounds in quantity. Projects done *in silico* can provide deep learning for future predictions and viability.

Organism as Lego set. The organism is a set of modular parts that can be pulled apart and reassembled to yield chimeras (novel constructs) with novel functions.^14^ The organism's genome may itself be treated as a module and endosymbiotically relocated into a new organism,^15^ with some sequences deactivated or eliminated to grow new organs, organelles, or organelle-like structures.

Organism as model system. The organism is a puzzle to solve in that the organism models a particular problem worth solving. The solution to the organism (or to the problem that the organism models) becomes a solution applicable to other organisms that are less experimentally tractable or more biologically complex.^16^ See, for instance, how sorghum functions as a model system for bioenergy crops with its photosynthetic efficiency.^17^ Although similar to the “expert” tag, model systems foreground the problem to be solved instead of the solution to be applied. In some cases, the rationale for selecting a specific model organism (or non-canonical model organism being used as a model) is explicit. In other cases, organisms are implicitly models, when they are positioned as tractable intermediaries toward studying more complex genomes.

Organism as sensor. The organism's metabolism is selectively altered to produce an indicator in response to a signal. It may be retooled to detect the presence of another species or a compound such as a pollutant or a diagnostic marker. Examples include testing for SARS-CoV-2,^18^, ^19^, ^20^ arsenic,^21^ and explosives.^22^ In some cases, indicator production is paired with a measure of survival (e.g., tryptophan concentration).

Organism as test-subject. The organism is a proxy for humans as a step toward precision-medicine, standing in for human subjects.^23^ The organism may either be chosen for its resemblance to some aspect of human physiology or “humanized” (e.g. humanized yeast,^24^ mice,^25^ rats^26^) with human DNA sequences or other discrete bits of human physiology. Test-subject organisms participate in elucidating human physiological mechanisms and thresholds relevant to toxicology, pharmacokinetics, disease progression, immune response, and transplant biology.^27^

Organism as stepping stone. These organisms are neither the intended product nor a model for another system, but a means to some other infrastructural end. These projects focus on building and refining technologies for future genomics research without being especially concerned with which organism helps get there. In these cases, the organism (still) matters because different organisms come with their own philosophical and logistical hurdles, care requirements, generation times, necessary tools and expertise.^28^ In these cases, even if the organism is more an afterthought than a central character, the organism's particular considerations still shape how technologies are developed.

Organism as commercial product. The organism is itself the final output and marketed as being able to accomplish a particular task. As a product, the organism (or group of organisms, in the case of probiotics) is made to exhibit some degree of consistent performance (i.e., through quality assurance testing) such that they become reliable commodities. A particular strain of the organism can be trademarked, distributed, and sold under a commercial name,^29^ or used to support other commercial exchanges.

## 3 Analysis

### 3.1 The organism bridges the transition from parts-to-whole synthesis

This heuristic helps us make sense of a nebulous field that originated in parts-based synthetic biology but now extends beyond it, not only in technical accomplishments but in scientific agendas, goals, and promises. Whole genomes have been synthesized in prokaryotes such as *Mycoplasma mycoides*^30^ and *Escherichia coli*,^31^ and the logical next step to synthesize larger, eukaryotic genomes is already underway. But the *Saccharomyces cerevisiae* 2.0 project (Sc2.0), as the largest example to date, exemplifies how wholes are divided into parts in practice (i.e. chromosomes and chunks of chromosomes), so that relying on part/whole distinctions to map the field is unreliable.^32^^,^^33^

At present, how genetic parts and genomic or organismal wholes relate, technically or conceptually, is not always clear or consistent. We see projects described in terms of their value for modifying a whole organism while still relying on conventional parts-based tools. Aside from precisely how this work happens in the lab, we want to know how researchers explain their projects in terms of whole-genome or whole-organism interventions since it is an important part of what synthetic genomics becomes. This is because language can shape ideas, including what stakeholders expect of the field, how scientists connect their work to funding opportunities, and how these will in turn shape ongoing technical developments.

### 3.2 Applying the heuristic to identify patterns

These tags allow us to look for patterns. For instance, are there correlations between clusters of tags, are these clusters more common in some sectors compared to others, and are certain tags associated with rationales that advance synthetic genomics? Our heuristic helps contextualize an organism, so that when a country has a rich history of using a particular organism, we can map the shifts in policy, funding calls, and roadmapping alongside the technologies developed. We can analyze the organism in its contexts, so that its role(s), its tools, and its applications can help us assess where the future of engineering biology is headed and anticipate the difficult questions related to social and regulatory concerns.

In addition, our tagging system can be applied to wild-type (i.e., no genetic modification) as well as partly modified organisms, so we can compare the subtle shifts that occur as a result of parts-based and whole-genome engineering. This also allows us to chart the evolution of a tag's use or the changes in an organism's roles depending on the context. For example, comparing the tags conferred to organisms with and without genetic modification or in academic versus industrial contexts can help us pinpoint what exactly differentiates organisms with synthetic genes and genomes—and how. This nuanced understanding would help us determine what drives the research involving genetically modifying organisms and their related dialogues, more so than using technological advancement alone to explain emerging biotechnologies.

We focus on the role of the organism because the organisms themselves are a constant in the transitions from parts to wholes, even though rationales for employing them might differ. Our interests are in understanding those differences, not just in terms of historical shifts of then versus now, but in a conceptual sense of how organisms are thought to be involved in a research project. Ultimately, we are asking questions about framing, about the roles that organisms are imagined into, because these imaginings will guide what organisms (and our relationships with them) become as researchers continue to engineer them.

### 3.3 Following the organism (and our relationships with them)

To date, parts-based synthetic biology has largely been characterized by organism agnosticism, a tendency to see only particular facets or functions of an organism without acknowledging or caring about the whole organism itself. In these instances, questions about which organism to use boil down to pragmatic concerns like life cycle, care needs, or cost. Early, high-profile, whole-genome projects seem to be characterized by an opposite extreme, in which the organism is valued not just for its technical properties but in light of its historical, social, and cultural significance. The synthetic yeast project is a good example. Researchers working with *S.cerevisiae* routinely frame their research in terms of yeast's ubiquity, utility, and friendliness, as demonstrated by the history of yeast genetics and the much longer history of brewing, baking, and winemaking. Here, the yeast is often described as being “charismatic,” with a “personality” that uniquely characterizes working relationships in the lab.^34^ We see similar attention to the historical, social, and cultural connections in other instances of genome-level work, including plants (e.g., potato, maize), insects (e.g., mosquito), animal (e.g., human, chicken), as well as less charismatic microbes (e.g., *Bacillus subtilis*). The organism is a prominent character in these studies, animating and animated by the research design.

Considering the field through this heuristic highlights working relationships among researchers, organisms, developing technologies, logics, roadmaps, promises, and risks. As we make out patterns among these roles, our hope is to identify how these relationships underwrite future innovations. Is it that organisms enrolled as chimeric Lego sets and used as pre-clinical test subjects are more likely to be taken up by mass media outlets? Are some tag combinations—such as “stepping stone” with “model system” tags or the combination of “expert” with “sensor” and “commercial product” tags—more likely to be funded than others, or more likely to develop new technologies and, with it, new organisms? Since engineering genomes has the potential to bring new types of organisms into the world, we want to be able to probe bigger questions about who gets to make new-ish organisms, in what contexts, for which goals, and at what expense.

Our point is not to make value judgments about organism use or organism redesign. This kind of line-drawing between acceptable and unacceptable uses is, appropriately, the jurisdiction of bioethicists. We are more interested in generating discussions about what collective wellbeing entails, given that engineering biology increasingly aims to construct new forms of life and new ways of living on (and sometimes off) this planet. Specifically, we want to ask questions about what a thriving future might look like without assuming an exclusively human biology is at stake.^35^ That said, we are wary of suggesting that all organisms are equally important, in ways that flatly and uncritically erase differences in how we relate to them and what we do with them. By centering the organism in conversations about engineering biology's future(s), we hope to foreground what living and working well with them means.

## 4 Future directions

We have developed this heuristic as part of an international project, “Future Organisms: Synthetic Genomics and Responsible Research in the UK, the USA, and Japan,” which attempts to open up discussions about what practices, policies, and potentials the future of biology could hold. Amongst the different foci, our interests lie with the creatures being designed and asked to perform certain roles in laboratory settings.

We are in the midst of conducting lab visits—in both academic and industry settings— along with interviews in the three countries being studied. By positioning ourselves as researchers interested in the organism, we hope to find commonalities with investigators within synthetic genomics as the subfield's boundaries become better defined.

## Declaration of competing interest

All authors declare that they have no conflicts of interest.
